# Mapping the global landscape of behavioral interventions for sarcopenia in older adults: implications for clinical practice and research prioritization

**DOI:** 10.1186/s12877-026-06970-5

**Published:** 2026-01-14

**Authors:** Chenhong Lin, Ling Pei, Changliu Xu, Xueying Chen, Jiajing Ma

**Affiliations:** 1 Department of Geriatrics, Shanghai Tenth People’s Hospital, Tongji University School of Medicine, 301 Yanchang Middle Road, Shanghai, 200072 China; 2 Department of Endocrinology and Metabolism, First Affiliated Hospital of Jinan University, Guangzhou, Guangdong 510632 China; 3 Department of Endocrinology, The First Affiliated Hospital, Sun Yat-sen University, Guangzhou, Guangdong 510080 China; 4 Department of Endocrinology, Guangdong Geriatrics Institute, Guangdong Provincial People’s Hospital, Guangdong Academy of Medical Sciences, Southern Medical University, Guangzhou, Guangdong 510080 China

**Keywords:** Sarcopenia, Behavior therapy, Clinical trials, Geriatric assessment, Implementation science

## Abstract

**Rationale:**

Sarcopenia is a significant health challenge for older adults. Behavioral interventions offer promising, accessible strategies, but the evidence base requires a comprehensive assessment.

**Aims and objectives:**

This study aimed to map the global landscape of clinical trials on behavioral interventions for sarcopenia, analyzing trial characteristics, geographic trends, and the translation of completed trials into publications.

**Methods:**

A systematic review of trials registered on ClinicalTrials.gov and the WHO International Clinical Trials Registry Platform (ICTRP) was conducted for trials registered up to 1 September 2024. Trials investigating non-pharmacological behavioral strategies for sarcopenia in adults ≥ 60 years were included. Data on design, sponsorship, location, and publication status were extracted and analyzed.

**Results:**

Among 171 identified trials, exercise training dominated (67.8%), with multicomponent and resistance training being the primary subtypes. Trials were concentrated in Asia (45.0%) and Europe (21.6%). Most were sponsored by academia (59.1%), enrolled ≤ 100 participants (73.1%), and used randomized designs (84.8%). Only 27 of 52 completed trials (51.9%) were published, with a median publication time of 14.5 months. The cumulative publication rate was 51.9% at 3 years post-completion.

**Conclusion:**

The research landscape is active but exhibits a pronounced focus on exercise, underrepresentation of other behavioral strategies, and significant delays in result dissemination. Coordinated efforts are needed to diversify interventions, ensure global representativeness, and improve the timely publication of findings to inform clinical practice.

**Supplementary Information:**

The online version contains supplementary material available at 10.1186/s12877-026-06970-5.

## Introduction

Sarcopenia is a condition marked by the gradual decline in muscle mass, strength, and functionality, presenting a significant health challenge for older adults. This condition is linked to a heightened risk of falls, fractures, disabilities, and increased mortality rates [1]. As the global demographic shifts toward older adults, sarcopenia has emerged as a pressing public health issue. The World Health Organization (WHO) has underscored the necessity of addressing sarcopenia within the framework of effective healthy aging strategies [2]. Behavioral interventions, which include exercise, dietary modifications, and lifestyle changes, offer promising avenues for intervention with potentially low risk and high accessibility [3, 4]. Prior research has demonstrated that modifications in behavior can notably influence muscle mass retention and overall health outcomes in older individuals [5]. However, many patients remain untreated or inadequately managed, indicating a critical gap in the healthcare delivery system, thus emphasizing the importance of further exploration of these strategies [6]. Clinical trials are essential for assessing the effectiveness and safety of such interventions. In accordance with the ethical principles of altruism and trust, the International Committee of Medical Journal Editors (ICMJE) mandates that all clinical trials be registered in a publicly accessible trial registry [7]. Registration helps disseminate information among clinicians, researchers, and patients, and increases public trust in medical science [8]. The high-capacity information contained in ClinicalTrials.gov provides a valuable opportunity to understand the landscape of trials for certain disease groups or indications. Timely and comprehensive analysis of clinical trials can present insights into the current medical research and identify research areas that are underappreciated [9]. Accordingly, this study provides a comprehensive overview of registered clinical trials (from ClinicalTrials.gov and ICTRP) on behavioral therapies for sarcopenia, aiming to characterize the research landscape and pinpointing existing evidence gaps.

## Methods

### Search strategy and data sources

We conducted a systematic search of clinical trials registered on ClinicalTrials.gov and the ICTRP, two widely recognized and comprehensive repositories of clinical trial information. ClinicalTrials.gov, maintained by the U.S. National Library of Medicine, is the largest public registry of clinical trials worldwide, while the ICTRP, established by the World Health Organization (WHO), provides a portal for searching clinical trials registered in primary registries around the globe.

### Data identification, and screening

For this study, ‘behavioral interventions’ were conceptually defined as strategies that primarily target the modification of a participant’s voluntary behavior, cognitions, and lifestyle habits through education, training, and motivation, without relying on the administration of specific pharmaceutical or nutraceutical products. This includes, for example, supervised or unsupervised exercise programs, dietary counseling aimed at whole-food intake, and lifestyle coaching. Interventions centered on the provision of oral nutritional supplements (ONS), specific medical foods, or drugs were excluded, as these are considered medical/nutritional treatments rather than behavioral modifications. For trials that included both behavioral and non-behavioral (e.g., supplemental or pharmaceutical) arms, the entire trial was retained for analysis if its primary purpose included the evaluation of a standalone behavioral intervention. This is because the registration characteristics of such a trial contribute directly to the landscape of behavioral intervention research. Trials were excluded only if all intervention arms necessitated the use of a non-behavioral component (e.g., a trial comparing ‘exercise + mandatory supplement’ vs. ‘supplement alone’), as the effect of the behavioral intervention could not be isolated for the purpose of our study.

Records of clinical trials that included “sarcopenia” in the “condition or disease” section and “behavioral” or “exercise” or “diet” or “training” or “physical activity” or “lifestyle” or “counseling” or “education” in the “intervention or treatment” section were extracted from the ClinicalTrials.gov database for trials registered before 1 September 2024. Concurrently, trials related to “sarcopenia” were retrieved from the ICTRP database, applying a filter to include only those with a behavioral intervention. Duplicate trials were identified by matching unique trial identifiers (e.g., NCT numbers) and trial titles across ClinicalTrials.gov and ICTRP. In cases of duplication, the record with the most complete data was retained. A total of 90 duplicates were removed. Trials were included in the analysis if they met the following criteria: (1) They were interventional, with at least one arm involving a behavioral-based therapy; (2) They focused on behavioral interventions for sarcopenia in older adults (≥ 60 years), including those with comorbidities (e.g., diabetes, obesity, cancer), provided that sarcopenia was a primary or co-primary focus. We excluded non-interventional studies, preclinical research, pediatric trials, and trials that exclusively involved pharmacological or nutraceutical interventions.; (3) Key data fields were available in the full-text registry.

### Data extraction and analysis

Trials were reviewed by two researchers (CL and LP) independently to screen eligibility, disagreements were resolved by consultation with a third reviewer (CX). The following information and data were extracted: the trial ID, registered title, study status, the sex and age of participants, enrollment size, location, study phase, the type and design of trials, intervention model, behavioral measures, primary purpose, funding information, registration date, primary completion date. As a mapping review, this study did not extract efficacy or safety data, nor did it assess the certainty of evidence or the risk of bias for individual trials, as its objective was to characterize the trial landscape rather than evaluate intervention effects. However, the publication status of completed trials was assessed as a measure of dissemination bias.

### Search for the publication of completed trials

We systematically searched PubMed and Web of Science for publications stemming from completed trials using the following strategy: trial registration number, official title, and acronym (if available). If multiple publications were identified for a single trial, the earliest article reporting primary outcomes was selected. Protocols, reviews, and preclinical studies were excluded. The search was finalized on 30 December 2024. If multiple publications were obtained from the same registry identifier, the earliest article reporting the primary outcome was selected. Research protocols, reviews, and preclinical data were excluded. The search for the publication was updated and finalized by 30 December 2024. The published time referred to the period between the primary completion date to the first-time primary result was published.

### Primary outcomes and multiple comparisons

The characteristics including age, sex, enrollment, trial status, location, sponsor type, and design properties including types of assignment, masking, intervention, and study purpose and phase of eligible trials were summarized. To facilitate a comparison of different behavioral intervention models, the trials were stratified into two groups according to intervention complexity: single-modality interventions and combined-modality interventions. Given the descriptive and mapping nature of this review, no quantitative synthesis (meta-analysis) was performed. Studies were grouped and summarized narratively and in tables according to their characteristics. No statistical pooling, assessment of heterogeneity, subgroup analyses, meta-regression, or sensitivity analyses were conducted. Results are presented as frequencies, percentages, medians, and interquartile ranges, as appropriate.

### Statistical analysis

Categorical variables were presented as frequencies and percentages, and compared using the Pearson chi-square test, or the Fisher exact test if the number of trials in any single category was less than 5. Continuous variables are represented as medians with inter-quartile ranges (IQRs). Cumulative publication rates after trial completion were analyzed using Kaplan-Meier. All statistical tests were performed using SPSS version 26.0 software (IBM Corp., Armonk, NY, USA), and a two-sided *P* value < 0.05 was considered statistically significant.

## Results

### Study selection

**Fig. 1:**
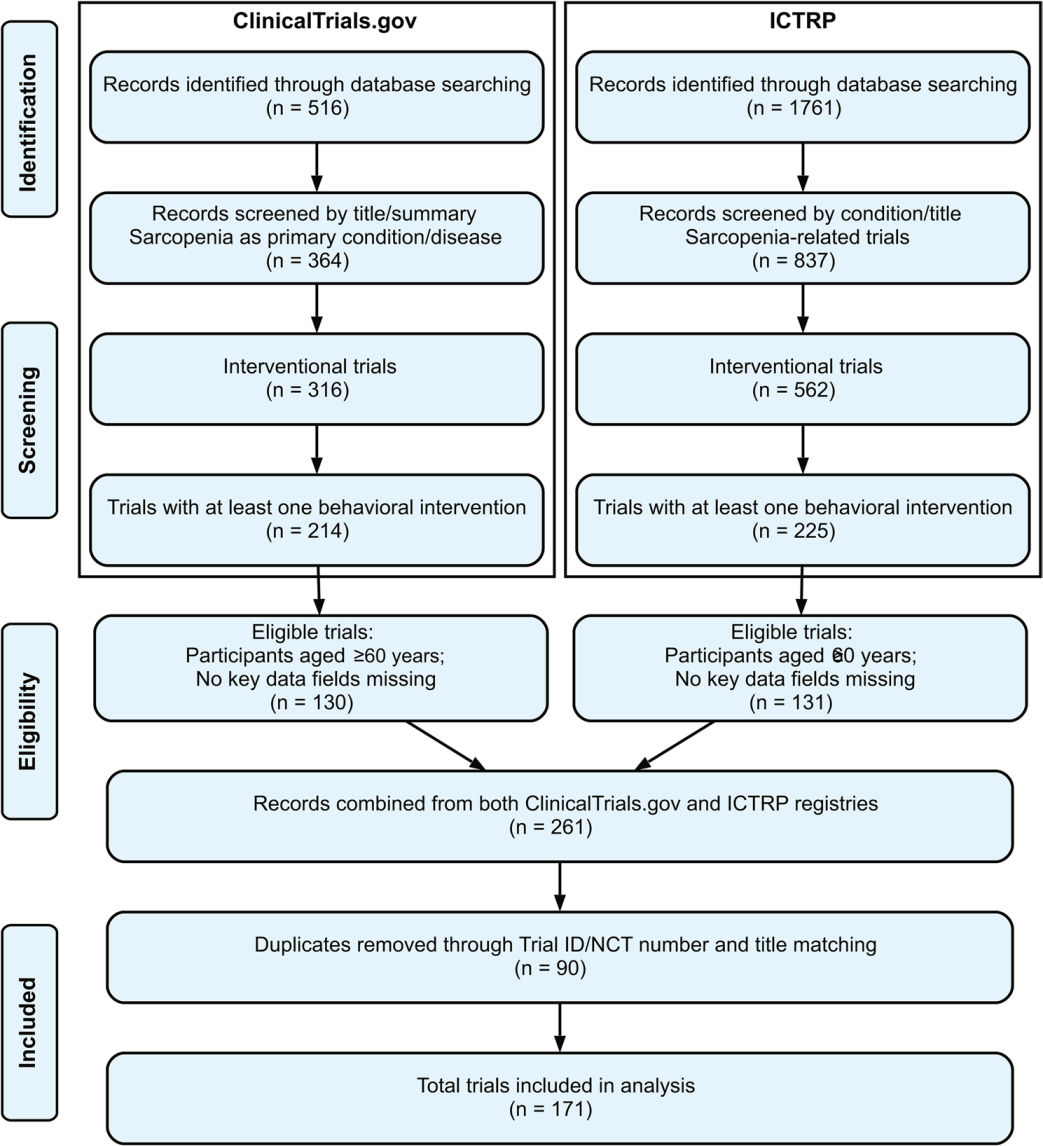
Flow chart of trials identification and screening

During the systematic selection process, studies were excluded at multiple stages based on pre-defined eligibility criteria. From ClinicalTrials.gov, 152 of 516 records were excluded for not focusing on sarcopenia, 48 were observational studies, 102 lacked a behavioral intervention arm, and 84 did not meet age criteria (≥ 60 years) or had missing key data. From the ICTRP, 924 of 1761 records were unrelated to sarcopenia, 275 were observational, 337 lacked a behavioral intervention, and 94 were excluded due to age or incomplete data. Additionally, 90 duplicates across registries were removed. The selection process is illustrated in Fig. 1. A list of all 171 included trials with their registry identifiers is provided in Supplementary Table S1.

### Overview of identified trials

**Fig. 2:**
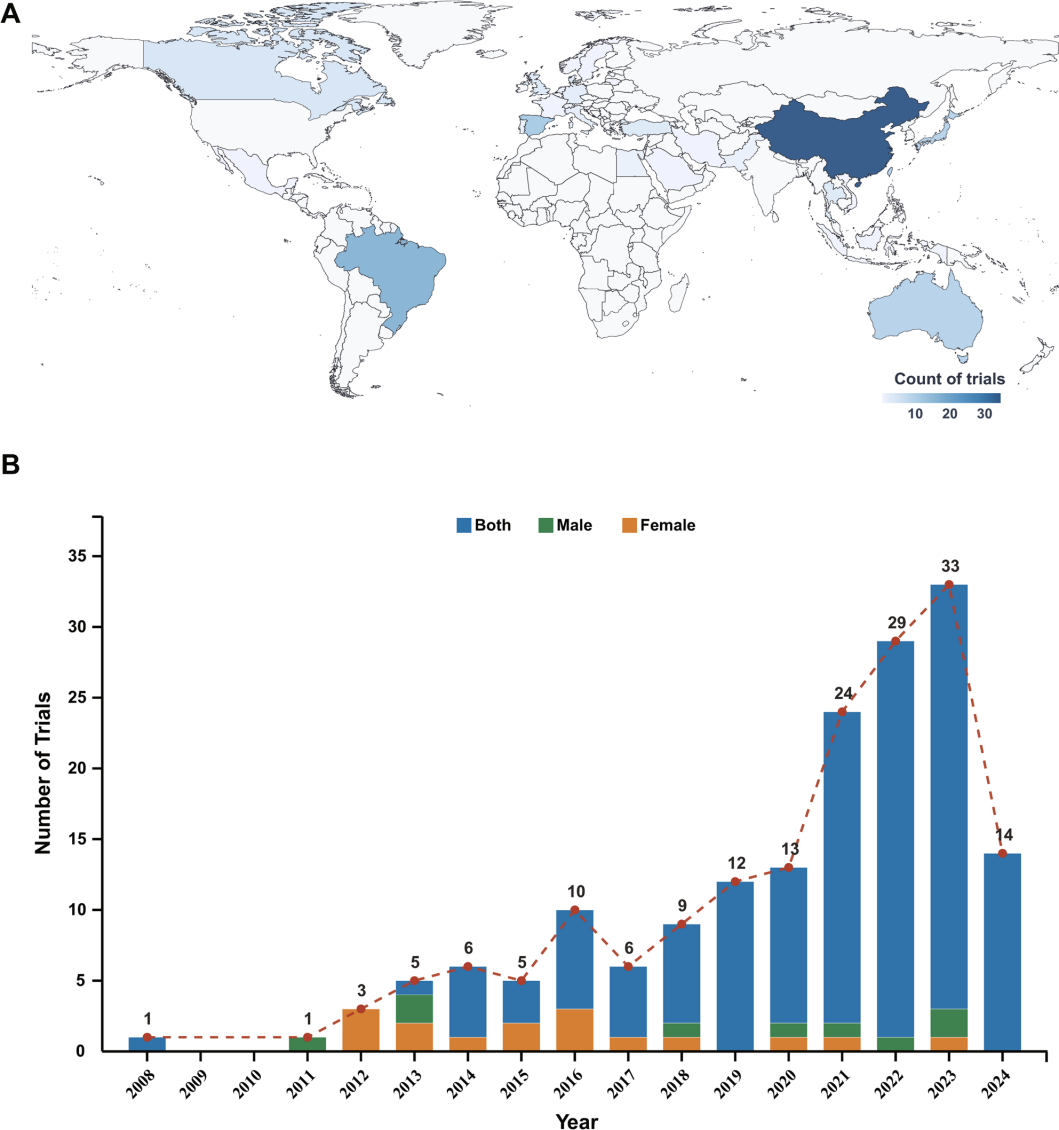
Distributions of eligible trials. (**A**) Global distribution of sarcopenia behavioral intervention trials. (**B**) Trends in sarcopenia behavioral intervention trials by participant sex

Our systematic search identified 171 registered interventional trials investigating behavioral strategies for sarcopenia in older adults (≥ 60 years). The global distribution was concentrated in Asia (45.0%), Europe (21.6%), and North America (16.4%) (Fig. 2A). Most trials (73.1%) were small-scale, enrolling ≤ 100 participants. Regarding trial status, 52.6% were not yet recruiting, 30.4% were completed, and 15.2% were actively recruiting. Academic institutions were the primary sponsors (59.1%), followed by hospitals (28.1%), while industry sponsorship was minimal (0.6%). The vast majority of trials (85.4%) included both sexes, with the annual initiation trend detailed in Fig. 2B. Methodologically, randomized designs (84.8%) using parallel assignment (83.6%) predominated. The primary purpose was most frequently classified as treatment (46.2%) or prevention (26.9%). Key characteristics are summarized in Table 1.

**Table 1:** Characteristics of behavioral intervention trials for sarcopenia in the older adults from ClinicalTrials.gov and the ICTRP

Variable	Category	*n* (%)
Location	Asia	77 (45.0)
	Europe	37 (21.6)
	North America	28 (16.4)
	South America	17 ( 9.9)
	Oceania	10 ( 5.8)
	Africa	2 ( 1.2)
Sample Size	*n* ≤ 100	125 (73.1)
	*n* > 100	46 (26.9)
Study Status	Completed	52 (30.4)
	Not Recruiting	90 (52.6)
	Recruiting	26 (15.2)
	Withdrawn	2 ( 1.2)
	suspended	1 ( 0.6)
Sponsor Type	Academic	101 (59.1)
	Government	6 ( 3.5)
	Hospital	48 (28.1)
	Industry	1 ( 0.6)
	Other	15 ( 8.8)
Sex Distribution	Both	146 (85.4)
	Female	16 ( 9.4)
	Male	9 ( 5.3)
Allocation Method	NA	8 ( 4.7)
	Non-Randomized	18 (10.5)
	Randomized	145 (84.8)
Intervention Model	Crossover	7 ( 4.1)
	Factorial	5 ( 2.9)
	Other	1 ( 0.6)
	Parallel	143 (83.6)
	Single Group	15 ( 8.8)
Masking Type	Double	24 (14.0)
	None (Open Label)	80 (46.8)
	Quadruple	4 ( 2.3)
	Single	57 (33.3)
	Triple	6 ( 3.5)
Primary Purpose	Basic Science	15 ( 8.8)
	Health Services Research	14 ( 8.2)
	Other	8 ( 4.7)
	Prevention	46 (26.9)
	Supportive Care	9 ( 5.3)
	Treatment	79 (46.2)
Trial Phase	Not Applicable	162 (94.7)
	Phase 1	5 ( 2.9)
	Phase 1/Phase 2	1 ( 0.6)
	Phase 3	2 ( 1.2)
	Phase 4	1 ( 0.6)
Primary Intervention Category	Combined Behavioral Interventions	34 (19.9)
	Dietary Behavior Change	11 ( 6.4)
	Exercise Training	116 (67.8)
	Lifestyle management	10 ( 5.8)
Publication Status (Completed Trials)	Not Published	25 (48.1)
	Published	27 (51.9)

Values are presented as n (percentage). Publication status is calculated only for completed trials

### Distribution of behavioral intervention type

**Fig. 3:**
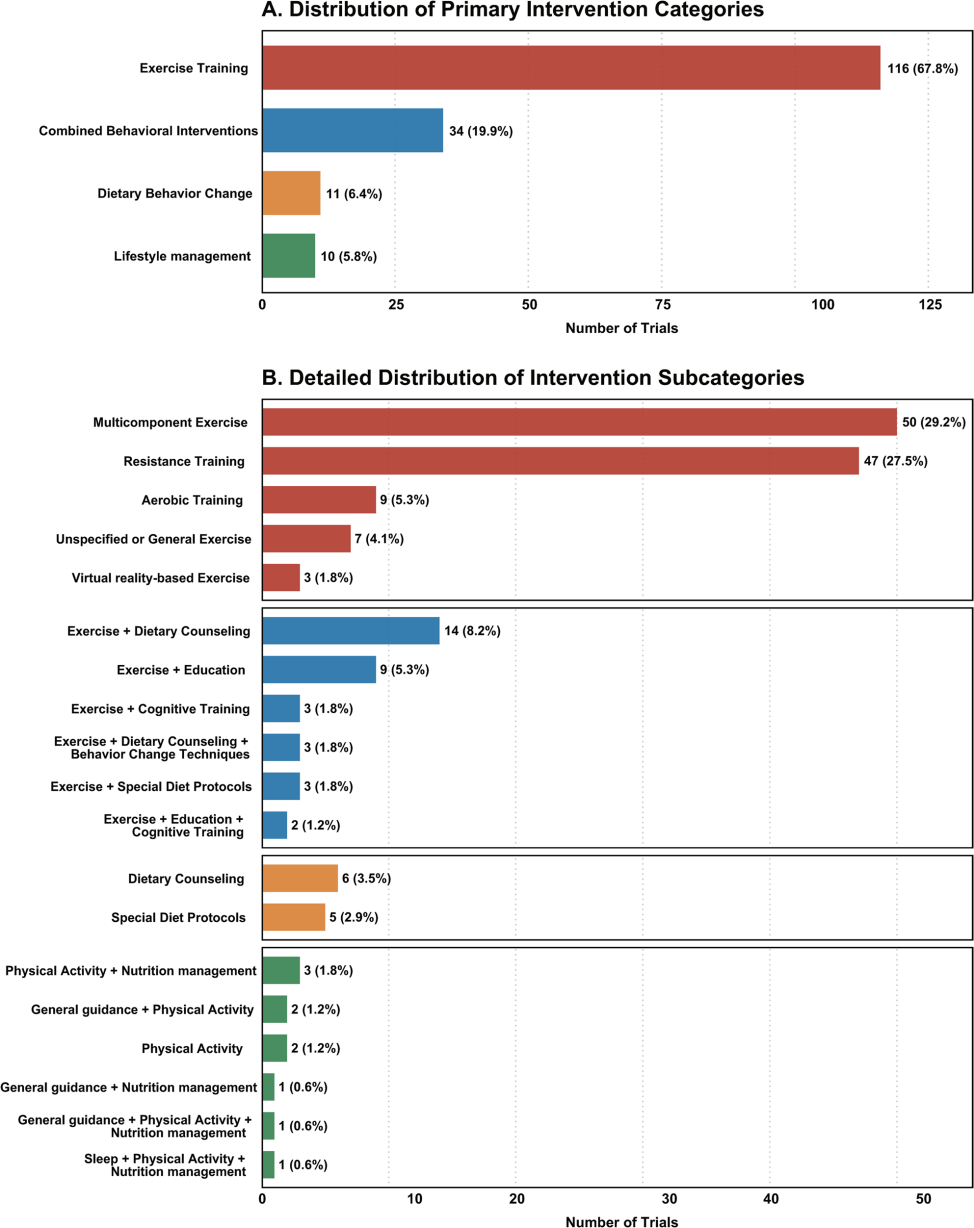
Distribution of behavioral intervention. (**A**) Distribution of primary intervention categories. (**B**) Detailed distribution of intervention subcategories

The types of behavioral interventions are detailed in Fig. 3. Exercise training constituted the majority of interventions (67.8%). Among these, multicomponent exercise (29.2%) and resistance training (27.5%) were the most common subtypes, followed by aerobic training (5.3%) and virtual reality-based exercise (1.8%). Combined behavioral interventions accounted for 19.9% of trials, with exercise plus dietary counseling (8.2%) being the most frequent combination. Isolated dietary behavior change interventions were less common (6.4%), primarily implemented through dietary counseling (3.5%) or special diet protocols (2.9%). Lifestyle management programs accounted for the remaining 5.8% of trials.

### Stratified analysis by intervention type

To further understand the landscape of behavioral interventions, we stratified the trials into two groups based on intervention complexity: single-modality interventions (exercise-only or diet-only, *n* = 127, 74.3%) and combined-modality interventions (interventions integrating multiple behavioral components, *n* = 44, 25.7%). A comparative analysis of their characteristics is presented in Table 2. A significant difference emerged in sample size distribution (*p* = 0.018). Combined-modality trials were substantially more likely to enroll over 100 participants (40.9%) compared to single-modality trials (22.0%). Conversely, the majority of single-modality trials (78.0%) had a sample size of 100 or fewer. The primary purpose also differed significantly (*p* = 0.005); while treatment was the main focus in single-modality trials (48.8%), combined-modality trials showed a greater emphasis on health services research (20.5% vs. 3.9%). No significant differences were observed in geographic distribution, sponsor type, or publication status among completed trials between single-modality group and combined-modality group.

**Table 2:** Characteristics of behavioral intervention trials for sarcopenia stratified by intervention type

Variable	Category	Single-modality *n* (%)	Combined-modality *n* (%)	*P* Value
Location	Africa	2 ( 1.6)	0 ( 0.0)	0.1544
	Asia	52 (40.9)	25 (56.8)	
	Europe	26 (20.5)	11 (25.0)	
	North America	26 (20.5)	2 ( 4.5)	
	Oceania	8 ( 6.3)	2 ( 4.5)	
	South America	13 (10.2)	4 ( 9.1)	
Sponsor Type	Academic	77 (60.6)	24 (54.5)	0.1039
	Government	6 ( 4.7)	0 ( 0.0)	
	Hospital	30 (23.6)	18 (40.9)	
	Industry	1 ( 0.8)	0 ( 0.0)	
	Other	13 (10.2)	2 ( 4.5)	
Sample Size	*n* > 100	28 (22.0)	18 (40.9)	**0.0180**
	*n* ≤ 100	99 (78.0)	26 (59.1)	
Study Status	Completed	39 (30.7)	13 (29.5)	0.9655
	Not Recruiting	66 (52.0)	24 (54.5)	
	Recruiting	19 (15.0)	7 (15.9)	
	suspended	1 ( 0.8)	0 ( 0.0)	
	Withdrawn	2 ( 1.6)	0 ( 0.0)	
Sex Distribution	Both	106 (83.5)	40 (90.9)	0.5297
	Female	13 (10.2)	3 ( 6.8)	
	Male	8 ( 6.3)	1 ( 2.3)	
Allocation Method	NA	7 ( 5.5)	1 ( 2.3)	0.3648
	Non-Randomized	11 ( 8.7)	7 (15.9)	
	Randomized	109 (85.8)	36 (81.8)	
Intervention Model	Crossover	6 ( 4.7)	1 ( 2.3)	0.9205
	Factorial	4 ( 3.1)	1 ( 2.3)	
	Other	1 ( 0.8)	0 ( 0.0)	
	Parallel	105 (82.7)	38 (86.4)	
	Single Group	11 ( 8.7)	4 ( 9.1)	
Masking Type	Double	18 (14.2)	6 (13.6)	0.3853
	None (Open Label)	55 (43.3)	25 (56.8)	
	Quadruple	4 ( 3.1)	0 ( 0.0)	
	Single	46 (36.2)	11 (25.0)	
	Triple	4 ( 3.1)	2 ( 4.5)	
Primary Purpose	Basic Science	13 (10.2)	2 ( 4.5)	**0.0050**
	Health Services Research	5 ( 3.9)	9 (20.5)	
	Other	8 ( 6.3)	0 ( 0.0)	
	Prevention	31 (24.4)	15 (34.1)	
	Supportive Care	8 ( 6.3)	1 ( 2.3)	
	Treatment	62 (48.8)	17 (38.6)	
Trial Phase	Not Applicable	122 (96.1)	40 (90.9)	0.1389
	Phase 1	2 ( 1.6)	3 ( 6.8)	
	Phase 1/Phase 2	1 ( 0.8)	0 ( 0.0)	
	Phase 3	2 ( 1.6)	0 ( 0.0)	
	Phase 4	0 ( 0.0)	1 ( 2.3)	
Publication Status	Not Published	18 (46.2)	7 (53.8)	0.7291
	Published	21 (53.8)	6 (46.2)	

Values are presented as n (%). Bold = *P* < 0.05 indicate a statistically significant difference between groups

Abbreviations: Single-modality = exercise-only or diet-only interventions, Combined-modality = interventions combining multiple behavioral components

### Publication outcome of completed trials

**Fig. 4:**
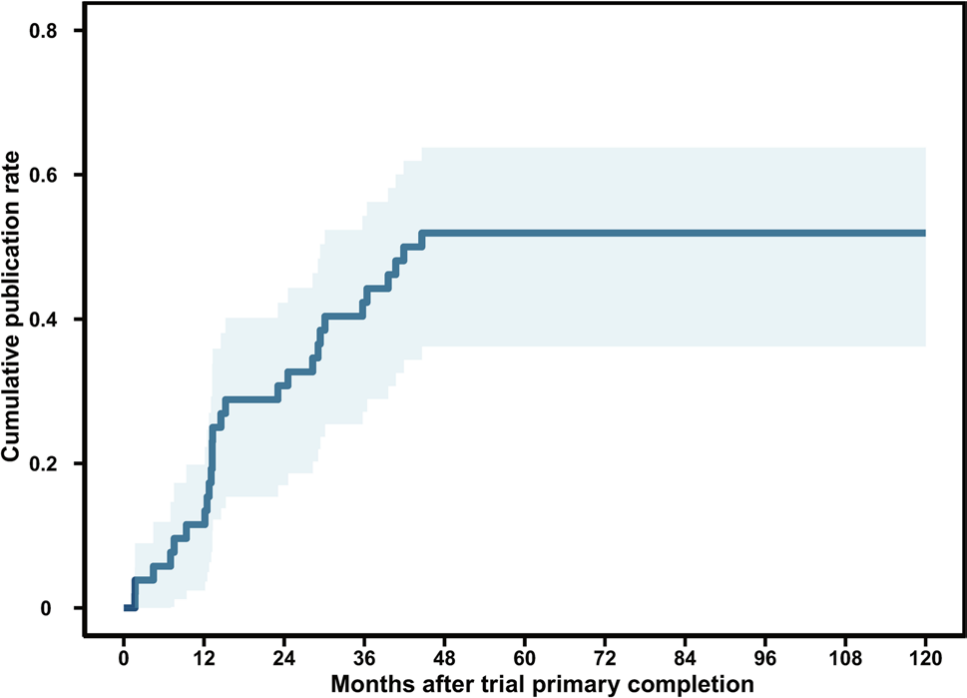
Cumulative publication rate curve after trial primary completion

Among the 171 identified trials, 52 (30.4%) were registered as completed. The publication status and timeliness of result dissemination for these completed trials are summarized in Fig. 4. By the final search date (30 December 2024), only 27 of the 52 completed trials (51.9%) had resulted in a peer-reviewed publication. The cumulative publication rate following the primary completion date was analyzed using the Kaplan-Meier method. Publication of results accrued over time, with cumulative rates reaching 15.4% within 1 year, 30.8% by 2 years, and 51.9% by 3 years following trial completion. The median time to publication was 14.5 months (IQR: 12.3–29.8). Notably, nearly half of the completed trials remained unpublished more than three years after completion, indicating a substantial delay in the dissemination of research findings.

## Discussion

Sarcopenia is a progressive condition marked by the loss of muscle mass and strength, particularly affecting the older adults and leading to serious health risks such as increased morbidity and mortality, falls, and a decreased quality of life [10, 11]. Addressing sarcopenia is crucial due to its association with adverse outcomes like diabetes, obesity, and reduced mobility, making it imperative to understand its implications for healthcare systems and the urgent need for targeted interventions and management strategies [12, 13]. This study provides a comprehensive mapping of the global clinical trial landscape for behavioral interventions in older adults with sarcopenia. By analyzing 171 registered trials, we identified distinct patterns in intervention types, geographic distribution, trial design, and the critical challenge of result dissemination. Our findings reveal that while research activity is growing, significant disparities and inefficiencies persist, which must be addressed to translate evidence into effective clinical practice.

The overwhelming dominance of exercise-based interventions (67.8%), particularly resistance and multicomponent training, aligns robustly with current international clinical guidelines for the management of sarcopenia [14, 15]. This consistency is encouraging and reflects a strong evidence base and consensus on the role of physical activity in preserving muscle mass and function. However, the relative underrepresentation of dedicated dietary behavior change interventions (6.4%) presents a notable disconnect from the equally compelling evidence supporting the critical role of adequate protein and energy intake in combating sarcopenia [5, 16]. While our operational definition excluded trials centered on Oral Nutritional Supplements (ONS) to focus on behavioral modifications, the low prevalence of trials investigating whole-diet approaches, dietary counseling, or special diets suggests a significant research gap. This focus on exercise, while justified, may have overlooked the synergistic potential of combined exercise and nutrition interventions, which have been shown to be more effective than either approach alone for certain outcomes [17]. Our stratified analysis offers further insight into the research paradigm. The finding that combined-modality trials were more likely to have larger sample sizes and a greater focus on health services research is logical, as these complex interventions often require robust designs to evaluate their real-world implementation and effectiveness [18]. Conversely, the preponderance of small-scale, single-modality trials, often focused on efficacy, suggests a field still building its foundational evidence. While these pilot and phase II-equivalent trials are essential, the low rate of industry sponsorship (0.6%) raises questions about the pathway for scaling up and translating these findings into widely accessible interventions. The geographic concentration of trials in high- and upper-middle-income regions, with minimal representation from Africa and South America, underscores a global equity gap in sarcopenia research. This is particularly concerning as these underserved regions are experiencing rapid demographic aging and may lack the resources to develop context-specific interventions based on this skewed evidence base [19].

A critical and concerning finding of our study is the suboptimal publication rate of completed trials. Only half of the completed trials were published within three years, with a median delay exceeding 14 months, leading to significant research resource waste and increasing the risk of publication bias [20]. This delay impedes the incorporation of the latest evidence into clinical guidelines and practice, potentially denying patients effective care. Underlying factors are likely multifaceted, such as insufficient funding for analysis and manuscript preparation, perceiving negative or null results as less publishable, and challenges in publishing complex behavioral intervention studies [21]. Publication bias is a well-known phenomenon where negative or inconclusive outcomes are less likely to be published [22]. The issue of under-reporting bias of trial results mainly depends on decisions taken by research sponsors and researchers, rather than journal editors rejecting submitted reports [20]. Therefore, improving the appropriate publication and dissemination of clinical trial results remains a pressing need. This issue underscores the urgent need for stricter enforcement of reporting mandates and the development of alternative dissemination pathways to ensure that the results of all registered trials contribute to the public knowledge.

Several limitations of our study should be acknowledged. First, our analysis relied on data from trial registries, although mandatory, can be incomplete, inaccurate, or not updated in a timely manner [21]. Second, our search, though systematic, may have missed some relevant trials that did not use our predefined keywords or were registered in smaller, non-ICTRP-affiliated registries. Third, as a mapping review of trial characteristics, we did not assess the risk of bias or the quality of the interventions within the trials, which limits any conclusions regarding the effectiveness of the strategies being investigated. Finally, our analysis could not capture patient-centered outcomes or long-term adherence data, which are crucial for understanding the real-world impact of these behavioral interventions.

## Conclusions

In conclusion, this landscape analysis reveals a vibrant but imbalanced field of research. The strong focus on exercise is aligned with evidence but may come at the opportunity cost of exploring nutritional and multimodal behavioral strategies. The geographic disparities and the substantial delay in publication of completed trials are major challenges that stakeholders, including funders, journals, and research institutions, must address collaboratively.

Clinically, our findings reinforce the importance of prescribing structured exercise, particularly resistance and multicomponent training, for older adults with or at risk of sarcopenia. For research, priorities should include: (1) designing and funding high-quality trials on dietary behavior change and combined interventions; (2) incentivizing trials in currently underrepresented world regions; and (3) implementing mechanisms to ensure the timely publication and dissemination of all trial results. By addressing these gaps, the research community can better generate the evidence needed to integrate comprehensive and effective behavioral strategies into routine geriatric care worldwide.

## Supplementary Information

Supplementary Material 1.

## Data Availability

The datasets generated during this study are available in the ClinicalTrials.gov and WHO ICTRP repositories. Derived data supporting the findings are available from the corresponding author on reasonable request.
